# Heterogeneity of psychophysiological stress responses in fibromyalgia syndrome patients

**DOI:** 10.1186/ar1863

**Published:** 2005-11-30

**Authors:** Kati Thieme, Dennis C Turk

**Affiliations:** 1 Department of Neuropsychology at the University of Heidelberg, Central Institute of Mental Health, J5, 68169 Mannheim, Germany; 2 Department of Anesthesiology at the University of Washington, NE Pacific Street, Seattle, WA 98195-6540, USA

## Abstract

Dysregulated psychophysiological responses have been observed in patients with fibromyalgia syndrome (FMS), although the results are inconsistent. Surface electromyographic (EMG), systolic and diastolic blood pressure, heart rate (HR), and skin conductance levels (SCLs) were continuously recorded at baseline, and during a series of stress and relaxation tasks in 90 FMS patients and 30 age and sex matched healthy controls (HCs). The patient sample demonstrated lower baseline EMG levels compared to the HCs on all tasks. In contrast, the patients displayed elevated HR and SCL (sympathetic vasomotor and sudomotor indices, respectively) during both stress tasks. A cluster analysis identified four psychophysiological response patterns: 63.3% of HCs showed increased muscle tension and stable cardiovascular responses; 34.8% of FMS patients showed a pattern of increased sympathetic vasomotor reactivity with stable sudomotor and reduced muscular response; 12.2% of FMS patients showed a pattern of increased sympathetic sudomotor reactivity connected with increased sympathetic vasomotor response and reduced muscular response; and, in contrast, 46.7% of FMS patients showed a pattern of parasympathetic vasomotor reactivity and reduced sudomotor as well as muscular response. The identification of low baseline muscle tension in FMS is discrepant with other chronic pain syndromes and suggests that unique psychophysiological features may be associated with FMS. The different psychophysiological response patterns within the patient sample support the heterogeneity of FMS.

## Introduction

Fibromyalgia syndrome (FMS) is defined as widespread pain combined with tenderness at 11 or more of 18 specific 'tender points' [1]. There is no consensus regarding the mechanisms underlying the set of symptoms reported by FMS sufferers. Additionally, several studies suggest heterogeneity in the diagnosis of FMS. For example, subgroup differences in biological variables such as positive antinuclear antibodies connected with features of connective tissue disease, interleukin 1β, interleukin-6, and tumor necrosis factor-alpha in skin (for example, see [2-4]), depression and cytokine abnormalities (for example, see [5,6]), and responses to pharmacological interventions (for example, see [7,8]) have been reported. Subgroups based on psychosocial responses have also been demonstrated [9]. Although abnormal responses to stress have been suggested to occur through a pathophysiological mechanism [10], research examining the influence of stress in FMS has yielded inconsistent results.

The majority of published studies evaluated the responses of the autonomic nervous system to physical stressful situations. This approach was used to test stress-reactivity as a potential cause of the maintenance of FMS symptoms. Several studies reported increased skin conductance levels (SCLs) [11], decreased heart rate (HR) variability [12], blood pressure (BP), and skin temperature [11] in response to physical stressors. These studies suggest an association between FMS and neurally mediated hypotension [13].

Although several studies that investigated surface electromyographic (EMG) activity failed to find differences between FMS patients and healthy controls (HCs) [14,15], others reported lower than average muscle tension levels in FMS patients in contrast to HCs during isometric exercises (for example, see [16,17]), following injection of hypertonic saline, or in antagonistic muscles [18,19]. Our own study used psychological stressors (for example, mental and social stress), and measured a complex physiological pattern consisting of EMG levels, BP, HR and SCLs. FMS patients displayed reduced muscle tension and increased heart rate. In contrast, HCs showed a modest HR response to stress. Furthermore, as a group the FMS patients demonstrated significant variability in stress reactivity and thus do not appear to be a homogeneous group when it comes to stress reactivity [20,21].

These results support the suggestion of autonomic response specificity [22,23] as an explanation for the different response patterns observed in FMS. Furthermore, the results suggest that patients who have the same diagnosis may have different psychophysiological response patterns.

The primary aim of the present study was to identify psychophysiological characteristics of FMS patients by examining BP, HR, SCLs, and surface EMG levels during baseline (BL) and stress conditions [23]. Based on the assumption of heterogeneity (for example, see [3,5,8,9]) and the studies by Qiao and colleagues. [11], Graven-Nielson and colleagues. [18], Sorensen and colleagues. [19], and Sprott and colleagues. [24], and our own study [20], we predicted enhanced autonomic system (for example, SCL, HR, and BP) responses, and lowered muscle tension (for example, EMG levels) and different psychophysiological response patterns within the FMS sample.

## Materials and methods

### Participants

Ninety female FMS patients recruited from a pain clinic, rheumatology outpatient departments and a hospital and thirty age and sex-matched HCs recruited from acquaintances of the investigated patients participated in the study. All patients met the American College of Rheumatology FMS criteria [1]. The exclusion criteria consisted of: inflammatory cause of the pain; neurological complications; pregnancy; concomitant severe disease; intake of muscle relaxants and opioids; major psychiatric disorder; and lack of language fluency. An institutional review board approved the study, which adhered to the Declaration of Helsinki and informed consent was obtained from all study participants.

Table 1 contains demographic and diagnostic information about the FMS patients and HCs. The sex-matched female HCs and FMS patients were comparable with respect to age and occupational status despite the fact that 25% of the FMS patients were receiving workers' compensation (chi(4) = 8.52, *p *= 0.074).

### Procedure

#### Clinical assessment

A physician performed an examination that included laboratory measures (for example, rheumatoid factor, antinuclear antibodies, erythrocyte sedimentation rate), and the evaluation of tender points (manual tender point survey [25]) on all FMS patients. The manual tender point survey was also performed on the HCs.

#### Psychophysiological assessment

**Figure 1:**
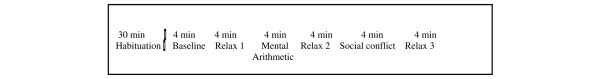
The design of the study.

Patients and HCs were instructed not to consume any analgesic or antidepressant medication for one day prior to their scheduled psychophysiological assessment. A 90 minute psychophysiological protocol was conducted subsequent to the medical and psychological assessments. The protocol consisted of 7 phases (Figure 1):

1. Adaptation (30 minutes): sitting quietly in a chair with eyes open.

2. Resting BL (4 minutes): sitting quietly with eyes open and to move as little as possible.

3. Relaxation (REL1, 4 minutes): pleasant music played through headphones with eyes closed.

4. Mental arithmetic (MA, 4 minutes): add 10 one-figure numbers in the presence of white noise (60 db). On 30% of the answers the participants were informed that their answer was 'incorrect' independent of their response. An additional white noise (60 db) stressor was delivered to the participants through headphones.

5. REL2: as described in 3.

6. Social conflict (SC, 4 minutes): discuss a SC from the list of unsolvable problems identified during the initial assessment [26].

7. REL3: as described in 3.

The stressor conditions were presented in a counter-balanced order to reduce any order effects.

Immediately following each phase, participants were asked to rate the intensity of their pain and perceived stress on an 11 point scale with the endpoints 'no pain' to 'very intense pain', and 'not at all stressful' to 'very stressful'.

### Psychophysiological recordings

Participants were seated and positioned in a straight back chair and were instructed to move as little as possible. A video camera located in the experimental room was activated throughout the psychophysiology protocol. All instructions were presented on a video screen.

EMG activity was recorded from the left and right m. trapezius according to the positioning recommended by Fridlund and Cacioppo [27]. BP was continuously monitored using an Ohmeda Finapres BP monitor (Datex-Ohmeda, Louisville, CO, USA). A MEDAT 6020 B Amplifier (Insight Instruments, Vienna, Austria) was used to record EMG, SCL, and HR. The presentation of the instructions, data acquisition, and data storage were computer-controlled. The sampling frequency of EMG signals was 3,000 Hz. The raw EMG was amplified by a factor of 100,000, passed through a bandpass filter (25 to 1,000 Hz), and integrated using contour-following integrators with a time constant of 70 ms.

BP was measured with a photoplethysmographic device on the fourth digit of the left hand (the accuracy of this procedure is ± 2 mmHg ± 0.25 kPa). A computer program that summed the digitized beat-by-beat waveforms averaged the sample time synchronized to the R-wave of the electrocardiogram, and divided them by the number of cardiac cycles. HR was determined with a multi-miniature transmitter using photoplethysmography of HR waveforms positioned on the tip of the fourth digit of the right hand. HR in beats per minutes was determined from this calculation [28]. SCL was measured through two electrodes in a multi-miniature sensor with a surface of 50.3 mm^2 ^on the second digit of the right hand about a constant current procedure of 4 μA [29,30]. All physiological measurements were continuously recorded like a 24 h BP monitoring.

### Data analysis

Data analyses were performed in several sequential steps. The first analyses assessed BL differences between the FMS and HC groups for the self-report and the psychophysiological variables. The second step examined differences in psychophysiological responses by the FMS and HC groups, using repeated measures analyses of variance (ANOVAs or ANCOVAs) depending on baseline differences with all six phases as within and the two groups as between factors. Significant ANOVA and ANCOVA effects were followed up by *post hoc t* tests in a third step. These *post hoc *analyses were used to calculate: (1) group differences over all six phases; (2) differences between the phases across both groups in order to test the stress induction; and (3) between group differences in stress reactivity. The stress and the relaxation phases were compared with the baseline for each group in the case of comparable baseline values. When there were baseline differences between the groups, ANCOVAs were performed. Significant effects were followed up by *post hoc t* tests to compare relaxation with stress phases. Bonferroni corrections were used to control for the large number of analyses (*p *< 0.005).

To identify psychophysiological response patterns we performed a z-transformation of all psychophysiological variables to create a common metric and thereby permit integration of the data as a fourth step. The z-transformations were performed for the overall sample to permit comparison of FMS patients and HCs. Finally, in order to examine the heterogeneity in physiological responding between and within the FMS and HC group, we performed a k-means cluster analysis [31]. The cluster analyses with z-transformed physiological variables permits identification of groups within the sample, the so-called 'psychophysiological patterns', and allows determination of the most stress-reactive physiological variable for each psychophysiological pattern. The cluster analysis used the means of all z-transformed physiological data collected during the BL, stress, and relaxation phases. The cluster analysis reveals an order of different psychophysiological variables with comparable z-scores. The variable with the highest z-score in each cluster characterizes the most reactive physiological system. The algebraic sign shows the direction of the stress response [32].

## Results

### Pain and stress response

#### Subjective pain ratings

As the BL pain ratings varied between the groups, an ANCOVA was performed. The results of this analyses revealed a significant main effect for groups (F(1,118) = 26.14, *p *< 0.001), indicating significantly higher pain ratings in the patient group than the HCs. A significant phase effect (F(5,114) = 7.79, *p *< 0.001) revealed that the pain ratings were higher in the stress than the REL phases (all *p*s < 0.01). The significant group × phase interaction (F(5,114) = 6.69, *p *< 0.001) indicated significant differences in pain ratings across phases for all FMS patients and the HCs controlled by covariate BL pain ratings.

#### Subjective stress ratings

As the BL values were different between groups (FMS and HC), an ANCOVA was performed with the BL scores as the covariate. The results of this analyses revealed that the group (F(1,118) = 52.27, *p *< 0.001), phase (F(5,114) = 32.43, *p *< 0.001), and a group × phase interaction (F(5,114) = 7.56, *p *< 0.001) were all statistically significant. FMS patients displayed significantly higher stress ratings than the HCs (all *p*s < 0.001; Table 2). The stress ratings were significantly higher in the stress compared to the REL phases (all *p*s < 0.001) controlled by covariate BL stress ratings.

### Psychophysiological data

#### Electromyographic changes

There were no significant differences between the left and right trapezius across any of the phases, thus combined means are reported. As the between group BL values were significantly different, an ANCOVA was performed. The group main effect (F(1,118) = 10.29, *p *< 0.005) was significant. FMS patients displayed significantly lower trapezius EMG activity than the HCs (*p *< 0.001). There were no statistically significant differences in EMG between BL, stress, and REL phases for either group when BL EMG was used as a covariate (Table 3).

#### Skin conductance level

A statistically significant phase effect (F(5,114) = 7.49, *p *< 0.001) indicated higher SCLs in the stress than the REL phases (all *p*s < 0.001). The significant group × phase interaction (F(5,114) = 3.84, *p *< 0.005) indicated that the FMS patients showed a significant increase from BL to REL1, and a significant increase from BL to MA and SC phases, in contrast to HC with no significant changes (Table 3).

#### Heart rate

HR showed a statistically significant group main effect (F(3,116) = 14.94, *p *< 0.001), indicating higher HR in FMS patients compared to the HCs (*p *< 0.001). The significant phase effect (F(5,114) = 15.08, *p *< 0.001) showed that HR increased in stress and decreased in REL phases across all FMS subgroups and the HCs (*p *< 0.001) (Table 3).

#### Blood pressure

A statistically significant phase effect (systolic blood pressure (SBP), F(5,114) = 30.92, *p *< 0.001; diastolic blood pressure (DBP), F(5,114) = 41.95, *p *< 0.001) indicated that SBP and DBP increased in stress and decreased in REL phases across the groups (*p *< 0.001). In contrast to SBP, DBP showed a statistically significant group × phase interaction (F(5,115) = 4.16, *p *< 0.005). The level of DBP in FMS patients decreased from BL to REL1 (*p *< 0.001), and increased from BL to MA and SC (all, *p*s < 0.001). The level of DBP increased in HCs from BL to MA and SC (all, *p*s < 0.005) (Table 3).

### Psychophysiological patterns

#### Characteristics of psychophysiological response patterns

The k-means cluster analysis (Table 4) yielded four clusters. The means of SBP (*p *< 0.001), DBP (*p *< 0.001), HR (*p *< 0.01), SCL (*p *< 0.001), and EMG (*p *< 0.001) were significantly different between the psychophysiological patterns (Table 4). The largest psychophysiological subgroup (n = 48) was characterized by a low autonomic and muscular response pattern with reduced SBP, DBP and HR and by very low SCLs and EMG levels. The low BP was the most reactive physiological system of the largest psychophysiological response pattern (for example, hypotensive reactivity). The second subgroup (n = 39) showed a high cardiovascular response pattern with elevated DBP, SBP, and HR, a moderate SCL response, and reduced EMG levels. The high BP was the most reactive physiological system. Elevated EMG levels and average responses in all other physiological data characterized the third subgroup (n = 22) with an enhanced muscular reactivity. The fourth pattern (n = 11) was characterized by a high autonomic and low muscular response pattern with high SCLs, increased cardiovascular variables and reduced EMG levels. The smallest pattern was characterized by SCL reactivity. The low autonomic and muscular stress response pattern with hypotensive BP reactivity was shown by 46.7% of FMS patients. The high cardiovascular response pattern with enhanced BP reactivity was shown by 37.8%, and the high SCL pattern with SCL reactivity by 12.2% of FMS patients. The high muscular response pattern with enhanced muscle reactivity was shown by 63.3% of HCs in contrast to only 3.3% of FMS patients (Table 5).

### Associations between psychophysiological response and self-reported measures

An ANOVA showed significant differences between the psychophysiological response patterns in pain (all F(3,117) = 7.46–12.31, *p *< 0.001) and stress ratings (F(3,117) = 4.06–13.25, *p *< 0.01) for all phases, as well as in the duration of pain (F(3,117) = 3.38, *p *< 0.05), although the latter two did not reach the Bonferroni-adjusted levels of statistical significance.

The high muscle response pattern showed significantly lower pain (*p *< 0.001), and the shortest duration of pain (*p *< 0.005) in contrast to the other psychophysiological response patterns (Table 6). There were no significant differences in pain and stress ratings between the other psychophysiological response patterns.

## Discussion

The results of this study demonstrate different physiological stress responses within FMS patients and between FMS patients and HC. The significant increase of BP, HR, and SCLs in the stress compared to the BL phase and the reduction in the REL phases indicates that stress and relaxation were induced, confirming the ecological validity of the stressors (compare to [33]).

Consistent with other studies [18,34], in the FMS patients, muscle tension at the BL and the experimental phases were significantly lower compared to the HCs. Moreover, although the FMS patients rated the SC and MA tasks as stressful, their muscle tension levels did not display elevations comparable to the HCs. It appears that neither mental stress nor pain intensity influence muscle tension in FMS patients.

Studies with P magnetic resonance imaging have reliably identified several abnormalities in the muscles of patients with FMS, including low levels of phosphocreatine and ATP at rest, low phosphorylation potential and total oxidative capacity, and a reduced number and size of mitochondria [23,35]. Additionally, the slower degradation of acetylcholine [36], which is involved in the production of corticosteroids and growth hormones [10,36], is an important regulator of muscle remodeling and performance [37]. Taken together, the results of the present study and other physiological studies [16-19,32] suggest that FMS is characterized by decreased muscle activity connected with an inability to respond adaptively to stress and relaxation. The reason for the decreased muscle activity in FMS does not appear to be only the result of physical deconditioning; ultrastructural changes in the muscle also appear to be involved [23,31]. Further investigations are needed to examine the interactions between muscle and the endocrine [37] and central nervous systems [38].

The four psychophysiological stress response patterns identified differentiated among the FMS sample and the HCs. The HCs were included to examine the diagnosis specificity of each of the FMS patterns. If the HCs had not been included in the analyses, it would not have been possible to interpret the three psychophysiological FMS patterns as we would not have known whether any of these three clusters reflected a normal stress reaction. The inclusion of the HCs permitted us to demonstrate that the FMS patterns were all completely different when compared to the response pattern showed by the HCs.

These differences are important because autonomic variables may be involved in the development and maintenance of chronic disease [21,22]. Flor and colleagues [39] found increased muscle reactivity in back pain patients following a psychological stress induction. Johannes and colleagues [32] found greater BP reactivity in patients with hypertension compared to HCs. The largest percentage of the FMS sample (46.7%) in the present study showed hypotensive reactivity within a stress response pattern that is characterized by decreased cardiovascular, SCL, and EMG values. The significantly lower BP suggests that the influence of parasympathetic reactivity may be extended during stress situations. Based on the the endocrine influence on the autonomic nervous system [40-42] and the central sensitization of FMS [43], a parasympathetic response pattern seems to be connected with the enhanced adrenocorticotrophic hormone production described in FMS (for example, see [10,36]).

The second largest response pattern (37.8%) of FMS patients was exemplified by increased BP reactivity. Increased cardiovascular stress responses and decreased SCLs and EMG levels also characterized this response pattern. The increased cardiac response suggests a tendency for higher peripheral sympathetic tones under stress. This psychophysiological response pattern replicates the results reported by Martinez-Lavin [44], who discussed FMS as a sympathetically maintained pain syndrome. Increased DBP appears to be connected to pain intensity (r = 0.32, *p *< 0.05). It is comparable to the stress response observed in rheumatoid arthritis patients who also showed enhanced BP reactivity [32]. Additional studies are necessary to confirm these results and to determine the mechanisms underlying these patterns.

The third largest response pattern of FMS (12.2%) was characterized by elevated SCL reactivity as sympathetic sudomotor reactivity and increased cardiovascular response as sympathetic vasomotor response as well as the FMS specific reduction in muscle tension. Patients with acute musculoskeletal injury also showed elevated sympathetic vaso- and sudomotor responses [45]. These sympathetic response patterns suggest that there is an interaction between cutaneous and vasomotor sympathetic neurons in response to acute musculoskeletal injury or to chronic pain. This is reflected by increased afferent input from sensitized nociceptors and other sensory neurons, resulting in alterations in autonomic function [45].

Johannes and colleagues [32] also found sympathetic and parasympathetic response patterns in the comparison of patients with hypertension, rheumatoid arthritis, and systemic lupus erythematosus. As the sympathetic and parasympathetic response patterns are present in FMS as well, it may be that these response patterns are relatively independent of the specific disease entity.

Moreover, the patients were medication-free only one day before the study. It is known that antidepressant medication affects autonomic nervous system activity. However, a comparison of the physiological stress responses of the subgroups of patients with (n = 20) and without (n = 60) antidepressant medication did not yield significant effects (parasympathetic DBP, t(46) = 0.08, *p* = 0.94); sympathetic stress response t(37) = 1.15, *p *= 0.26). Antidepressant use was not associated with altered stress response. Further, the physiological response did not show significant differences between patients with and without antidepressant use.

## Conclusion

The results of this study support the suggestion of heterogeneity of the mechanisms involved in FMS. They suggest further that differential treatment strategies matched to different patterns may be appropriate [46,47].

Although the overall sample size for the patient group appears reasonable, subdividing the total sample into four psychophysiological patterns produced relatively small groups. Thus, the interpretation of the results of the cluster analysis on the subgroups must be treated with caution. Research with larger samples is needed to replicate autonomic response specificity observed in the different psychosocial subgroups. Moreover, studies are needed to compare the psychophysiological reactivity in FMS with other chronic pain conditions to determine if the patterns observed are unique to FMS or are characteristic of chronic pain. Further, future research is needed to test endocrine predictors of stress reactivity in FMS to determine if the endocrine reaction is the cause or the consequence of FMS.

## Abbreviations

BL = baseline phase; BP = blood pressure; DBP = diastolic blood pressure; EMG = electromyographic activity; FMS = fibromyalgia syndrome; HC = healthy control; HR = heart rate; MA = mental arithmetic phase; REL = relaxation phase phase; SBP = systolic blood pressure; SC = social conflict phase; SCL = skin conductance level.

## Competing interests

The authors declare that they have no competing interests.

## Authors' contributions

KT: recruitment of the patients, organization and realization of the experimental design, statistical analyses, preparation of the manuscript. DCT: statistical analyses, preparation of the manuscript.

## Figures and Tables

**Table 1:** Demographic and clinical variables of the fibromyalgia syndrome patients and healthy controls

	FMS (n = 90) (mean ± SD (range))	HC (n = 30) (mean ± SD (range))
Age (years)	48.17 ± 10.32 (21–68)	48.22 ± 9.02 (22–65)
Duration of pain (years)	9.28 ± 9.23 (0.5–45)	
Painful regions	6.80 ± 2.05 (3–12)	
Number of tender points	16.09 ± 3.33 (14–18)	2 ± 0.33 (0–4)
Mean tender points pain severity^a^	5.89 ± 2.27 (2–10)	3.13 ± 1.06 (0–3)
Use of antidepressive medications (number)	0.36 ± 0.52 (0–3)	0.21 ± 0.03 (0–2)
Occupational status	N (%)	N (%)
Working	39 (43.3)	17 (56.8)
Unemployed	20 (22.2)	9 (30.0)
Workers' compensation	24 (26.7)	0 (0.0)
Retired	6 (6.7)	2 (6.6)
Student	1 (1.1)	2 (6.6)

^a^Visual analogue scale ranges from 0 = no pain to 10 = most intense pain. FMS, fibromyalgia syndrome; HC, healthy control; SD, standard deviation.

**Table 2:** Comparison of self-reported pain and stress between fibromyalgia syndrome patients and healthy controls

Phase	FMS (N = 90)		HC (N = 30)		*p *value
	Mean	SD	Mean	SD	

Pain					
Baseline	4.70	(2.30)	0.00	(0.00)	<0.001
Relax 1	4.75	(2.14)	0.00	(0.00)	<0.001
Arithmetic	5.60	(2.19)	0.07	(0.25)	<0.001
Relax 2	5.33	(1.70)	0.00	(0.00)	<0.001
Conflict	5.63	(2.03)	0.34	(0.71)	<0.001
Relax 3	5.40	(1.79)	0.23	(0.50)	<0.001
Stress					
Baseline	2.65	(0.92)	1.17	(0.79)	<0.001
Relax 1	2.22	(0.85)	0.17	(0.38)	<0.001
Arithmetic	4.70	(2.01)	2.37	(0.72)	<0.001
Relax 2	3.10	(1.10)	0.10	(0.31)	<0.001
Conflict	5.60	(1.88)	3.33	(1.03)	<0.001
Relax 3	3.04	(0.98)	0.57	(0.68)	<0.001

FMS, fibromyalgia syndrome; HC, healthy controls; SD, standard deviation.

**Table 3:** Differences in physiological variables between fibromyalgia syndrome patients and healthy controls

Phase	FMS (N = 90)		HC (N = 30)		*p *value
	Mean	SD	Mean	SD	

EMG					
Baseline	8.97	(4.75)	14.52	(5.12)	<0.001
Relax 1	8.77	(4.83)	15.83	(5.23)	<0.001
Mental arithmetic	10.44	(5.89)	17.10	(9.07)	<0.001
Relax 2	8.91	(4.91)	17.60	(9.44)	<0.001
Social conflict	9.59	(5.59)	17.04	(8.92)	<0.001
Relax 3	9.63	(6.33)	18.50	(10.51)	<0.001
SCL					
Baseline	1.81	(1.70)	1.65	(0.64)	ns
Relax 1	1.89	(1.78)	1.59	(0.62)	ns
Mental arithmetic	2.81	(2.65)	1.92	(0.74)	<0.005
Relax 2	2.31	(2.01)	1.76	(0.69)	ns
Social conflict	2.76	(2.11)	1.79	(0.72)	<0.005
Relax 3	2.41	(2.15)	1.69	(0.71)	ns
HR					
Baseline	77.07	(11.22)	68.56	(22.49)	ns
Relax 1	75.45	(10.54)	65.68	(21.11)	<0.005
Mental arithmetic	78.32	(13.64)	75.85	(24.72)	ns
Relax 2	73.36	(10.86)	66.47	(19.55)	ns
Social conflict	77.88	(10.64)	66.85	(20.88)	ns
Relax 3	73.96	(9.59)	62.79	(18.11)	<0.005
SBP					
Baseline	133.5	(19.03)	132.9	(13.40)	ns
Relax 1	131.4	(16.65)	130.4	(11.58)	ns
Mental arithmetic	141.7	(19.52)	140.8	(14.35)	ns
Relax 2	128.1	(13.20)	124.9	(21.00)	ns
Social conflict	146.5	(15.09)	139.7	(18.29)	ns
Relax 3	135.6	(14.31)	131.9	(23.38)	ns
DBP					
Baseline	78.43	(13.89)	77.10	(8.83)	ns
Relax 1	76.23	(8.27)	76.24	(7.79)	ns
Mental arithmetic	81.11	(10.80)	82.51	(10.83)	ns
Relax 2	76.82	(14.54)	73.37	(7.61)	ns
Social conflict	85.81	(9.33)	83.74	(10.29)	ns
Relax 3	77.57	(7.75)	78.04	(9.60)	ns

DBP, diastolic blood pressure; EMG, electromyographic activity; FMS, fibromyalgia syndrome; HC, healthy controls; HR, heart rate; ns, not significant; SBP, systolic blood pressure; SCL, skin conductance level.

**Table 4:** Z-scores of the four psychophysiological response patterns

Variable	Physiological response pattern				F	*p *value
	1	2	3	4		

Z-DBP-M	-0.69	**0.70**	0.18	0.48	31.044	<0.001
Z-SBP-M	**-0.71**	0.65	0.14	0.57	38.079	<0.001
Z-HR-M	-0.02	0.45	-0.04	0.21	4.612	0.005
Z-SCL-M	-0.43	-0.10	-0.18	**2.71**	131.298	<0.001
Z-EMG-M	-0.22	-0.35	**1.34**	-0.40	43.799	<0.001

Bold text indicates the highest values of each group. DBP, diastolic blood pressure; EMG, electromyographic activity; HR, heart rate; SBP, systolic blood pressure; SCL, skin conductance level.

**Table 5:** Psychophysiological response patterns in fibromyalgia syndrome patients and healthy controls

Group	PRP^1^	HC (N = 30)		FMS (N = 90)		Total	
		N	%	N	%	N	%

1	Low BP	6	20.0	42	**46.7**	48	40.0
2	High BP	5	16.7	34	**37.8**	39	32.5
3	High EMG	19	**63.3**	3	3.3	22	18.3
4	High SCL	0	0.0	11	12.2	11	9.2

Total		30	100.0	90	100.0	120	100.0

Bold text indicates the highest values of each group. ^1^Psychophysiological response patterns: Low BP: low systolic blood pressure (SBP), diastolic blood pressure (DBP), heart rate (HR), skin conductance level (SCL) and electromyographic activity (EMG); High BP: enhanced SBP, DBP, HR as well as moderate SCL and low EMG; High EMG: enhanced EMG as well as stable physiological response; High SCL: enhanced SCL as well as enhanced BP, moderate HR and reduced EMG. HC, healthy control; FMS, fibromyalgia syndrome.

**Table 6:** Self-reported pain and stress during the experiment and significant differences between psychophysiological response patterns

Phase	Low BP (N = 48)		High BP (N = 39)		High EMG (N = 22)		High SCL (N = 11)		Significant difference	
	Mean	SD	Mean	SD	Mean	SD	Mean	SD	F	*p*

Pain										
Baseline	3.36	(2.46)	3.73	(2.53)	1.00	(2.05)	5.17	(3.19)	7.46	<0.001
Relax 1	3.18	(2.45)	3.39	(2.32)	0.92	(1.90)	5.06	(3.06)	7.52	<0.001
Arithmetic	4.02	(2.86)	4.61	(2.56)	0.95	(1.93)	6.11	(3.06)	11.02	<0.001
Relax 2	3.64	(2.50)	4.26	(2.48)	0.95	(2.04)	5.50	(2.84)	10.12	<0.001
Conflict	3.88	(2.51)	4.69	(2.67)	0.92	(2.02)	6.06	(3.00)	12.12	<0.001
Relax 3	4.15	(2.56)	4.43	(2.31)	1.00	(2.00)	6.17	(3.10)	12.30	<0.001
Stress										
Baseline	2.40	(1.18)	2.31	(0.95)	1.58	(0.96)	3.11	(1.96)	4.06	0.009
Relax 1	1.79	(0.99)	2.00	(1.13)	0.47	(0.84)	3.11	(1.96)	13.25	<0.001
Arithmetic	4.40	(2.32)	4.84	(1.84)	3.00	(1.20)	5.94	(2.21)	5.52	0.001
Relax 2	2.57	(1.55)	2.69	(1.37)	0.53	(1.07)	3.44	(1.88)	12.81	<0.001
Conflict	5.25	(1.94)	6.08	(2.09)	3.84	(1.77)	6.56	(2.24)	6.41	0.001
Relax 3	2.83	(1.53)	2.62	(1.23)	1.00	(1.11)	3.33	(2.23)	8.75	<0.001
Duration of pain	8.35	(10.61)	7.16	(6.80)	1.33	(3.24)	5.33	(2.54)	3.38	0.022

BP, blood pressure; EMG, electromyographic activity FMS, fibromyalgia syndrome; HC, healthy controls; SCL, skin conductance level; SD, standard deviation.
